# Stimulation with a class A CpG oligonucleotide enhances resistance to infection with feline viruses from five different families

**DOI:** 10.1186/1297-9716-43-60

**Published:** 2012-08-20

**Authors:** Céline Robert-Tissot, Vera L Rüegger, Valentino Cattori, Marina L Meli, Barbara Riond, Peter F Moore, Monika Engels, Marco Franchini, Regina Hofmann-Lehmann, Hans Lutz

**Affiliations:** 1 Clinical Laboratory, Vetsuisse Faculty, University of Zurich, Winterthurerstrasse 260, CH-8057, Zurich, Switzerland; 2 Department of Pathology, Microbiology and Immunology, 3315 Vet Med 3A, School of Veterinary Medicine, University of California, Davis, CA, 95616, USA; 3 Institute of Virology, Vetsuisse Faculty, University of Zürich, Winterthurerstrasse 266a, 8057, Zürich, Switzerland

## Abstract

Domestic cats are commonly affected by viral pathogens that induce lengthy infections with fatal outcomes. Prevention of viral propagation is of primordial importance in shelters and catteries, where cats from different backgrounds have narrow contacts. Oligonucleotides (ODN) containing cytosine-phosphate-guanosine motifs of class A (CpG-A) are highly potent synthetic inducers of innate antiviral mechanisms. The aim of this study was to test their ability to modulate innate immune responses and prevent viral replication as stand-alone agents in the domestic cat. CpG-A stimulation of feline peripheral blood mononuclear cells (PBMCs) enhanced their proliferation, increased the presence of co-stimulatory molecules on their surface and influenced their gene expression profiles in an antiviral orientation. Incubation of the supernatants of CpG-A stimulated PBMCs with feline cell lines of epithelial and fibroblastic origin induced expression of the antiviral myxovirus resistance (Mx) gene in these target cells, which also showed enhanced resistance to feline viruses from five distinct families, namely *Coronaviridae*, *Herpesviridae*, *Caliciviridae*, *Parvoviridae*, and *Retroviridae*. Most importantly, subcutaneous administration of CpG-A in domestic cats systemically increased the expression of Mx, reaching maximal levels within 24 h. Plasma from treated cats could furthermore inhibit viral replication *in vitro*. Altogether, our data highlight the promising potential of CpG-A to induce a preventive antiviral state in the cat and to protect feline populations against a broad range of virus infections.

## Introduction

Feline viruses are of particularly opportunistic character. In order to adapt to the solitary way of life of ancestral felids, these pathogens have acquired elaborate means to persist within their host population over the course of time. The infection of new hosts upon the rare contact between individuals was evolutionarily assured by very efficient transmission strategies and the induction of latent, chronic and/or asymptomatic infections. Often kept in multi-cat households and placed in catteries or shelters, today’s domestic cat is consequently particularly susceptible to viral infections [1,2]. The close proximity and high social contact rate of animals with different vaccination states in these stressful environments further increases the risk of infection. Moreover, the strong antigenic variability of several common feline viruses including the feline calicivirus (FCV), the feline coronavirus (FCoV) and the feline leukemia virus (FeLV) support escape from immune responses and lower the efficacy of currently available vaccines [3]. The availability of complementary prophylactic strategies could help protect pet cats in environments with high infectious pressure from long diseases and/or death caused by infections with fatal viruses.

A promising addition to vaccination is the manipulation of innate immunity. Innate pathogen recognition relies on a set of sensory molecules, the Toll-like receptors (TLRs), which enable the immediate reaction of specific immune cells to pathogen “danger signals”, the so-called pathogen-associated molecular patterns (PAMPs) [4]. Due to their abundance in all bacterial as well as some viral genomes, oligodeoxynucleotides (ODN) containing unmethylated cytosine–phosphate–guanosine (CpG) motifs are effectively recognized as PAMPs by the vertebrate innate immune system [5]. Response to CpG ODN stimulation is conferred through TLR9, expressed mainly in the intracellular compartments of human B cells and plasmacytoid dendritic cells (pDCs) [6,7]. Alarmed TLR9 is the initial instigator of gene expression profiles that strongly support antiviral mechanisms: upregulation of costimulatory molecules major histocompatibility complex (MHC) II, B7.1 and B7.2 on the surface of stimulated cells provides them with a stronger antigen presenting potential [8,9] and production of cytokines such as type I interferon (IFN), interleukin (IL)-12, IFNγ, IL-6 and tumor necrosis factor (TNF)α, contribute to providing an optimal immune environment for the development of innate and adaptive responses against intracellular pathogens [10,11]. Probably the most important antiviral property of CpG ODN resides in their potential to stimulate the production of high amounts of type I IFN by pDCs [12]. This family of cytokines, which includes IFNα, IFNω and IFNβ, has been shown to considerably enhance natural killer (NK) cell cytotoxicity [13], promote differentiation, maturation and immunostimulatory functions of monocytes and DCs [14], induce B cell production of immunoglobulin [15] and T-helper (Th)1 differentiation of T cells [16,17]. Moreover, upon binding to their ubiquitously distributed receptor, type I IFNs effectively induce the synthesis of various intracellular proteins which interfere with the replication of a broad range of viruses [18]. The myxovirus-resistance protein (Mx) GTPase is a well-studied example of these intracellular antiviral effectors. This enzyme is known to be directly regulated by the type I IFN, and its detection is readily used as marker for upregulation and biological activity of this cytokine family [19].

Distinct classes of ODN have been shown to induce differential immune responses [20]. Class A CpG ODN (CpG-A) consist of CpG motifs in a phosphodiester core, flanked on both ends by phosphorothioate poly (G) sequences. CpG ODN of this class are characterized by their potential to both induce massive type I IFN secretion by pDCs [12] and increase NK cytotoxicity [21], rendering them ideal candidates as prophylactic enhancers of innate immunity to viral infections. Conversely, class B CpG ODN (CpG-B), which encode multiple CpG motifs on a phosphorothioate backbone, promote monocyte maturation and B cell activation, thus substantially supporting the development of humoral immune responses [22-24]. Both CpG-A and CpG-B have indicated immunostimulatory properties in immune cells of mice, primates and many domestic species *in vitro*[25-31], while *in vivo* studies in outbred animals have mainly been carried out with CpG-B [24,32,33]. Concerning the cat, specifically synthesized CpG-A molecules were recently shown to induce IFNγ [34]; CpG-B molecules indicated the capability to induce proliferation of feline cells [35], and CpG-B-adjuvanted allergen indicated potential for immunotherapy in a feline asthma model [36]. Although class C [37,38] and class P ODN [39] were developed in more recent years in an effort to combine the advantageous effects of both CpG-A and CpG-B and increase immunogenicity, CpG-A remain the strongest inducers of type I IFN described to date.

The potential of CpG ODN as prophylactic stand-alone inducers of innate defense mechanisms has been the subject of only few studies. Most works in this field initially described protection of mice against bacterial [40-44] and parasitic [45-47] infections. More recently, induction of resistance to viral infections was shown also in mouse models for Herpes Simplex Virus [48], neurotropic arenavirus [49], foot and mouth disease virus [50] and Vaccinia virus [51]. With exception of the latter, all these studies were carried out with CpG-B. In an outbred species, partial antiviral protection has only been described in two studies so far, in which reduced shedding of herpes and parainfluenza viruses was observed in lambs after administration also of a CpG-B [52,53]. To our knowledge, prophylactic antiviral potential of CpG-A has not yet been described in outbred animals.

We carried out a series of experiments with the objective to characterize both the immunomodulatory and antiviral properties of CpG-A in the domestic cat. *In vitro*, the prototype of CpG-A, ODN 2216 [12], strengthened the antiviral qualities of feline immune cells and stimulated their production of soluble molecules that inhibited the replication of five different families of viruses: *Coronaviridae*, *Herpesviridae*, *Caliciviridae*, *Parvoviridae*, and *Retroviridae. In vivo*, ODN 2216 induced a systemic antiviral state.

## Materials and methods

### Animals and ethics statement

Fourteen male castrated SPF cats divided in four different age groups were used in this study: group 1 (c01-c04, 10 weeks), group 2 (c05-c08, 1.5 years), group 3 (c09-c12, 7 years) and group 4 (c13 and c14, 14 years). Cats c13 and c14 from group 4 originated from the same litter; all other individuals were not related to each other. The *in vivo* experiments were conducted when cats c05-c08 were 3 years of age. All animals were purchased from Liberty Research Inc. (Waverly, NY, USA) and their SPF status was verified as previously described [54]. This study was carried out in strict accordance with regulations of the Swiss law for animal protection (SR 445.1). The Veterinary Office of the Swiss Canton of Zurich officially revised the protocol and approved the study (Permit no. TVB 99/2007 and TVB 100/2007). The animals were housed in groups in an animal-friendly barrier facility under optimal ethological conditions [55]. For blood collections and injections, the cats were sedated with a combination of ketamin and midazolam, and all possible efforts were made to minimize stress and suffering.

### Feline PBMC isolation, cell lines, ODNs, cell culture and cell viability assay

Feline PBMCs were isolated from EDTA-anticoagulated whole blood by Ficoll-Hypaque density gradient centrifugation using a standard protocol [56]. Purified cells were counted prior to their utilization in the different experiments using the Sysmex XT 2000iV (Sysmex, Norderstedt, Germany) as described previously [57], and cultured in RPMI 1640 with Glutamax I (Gibco®, Invitrogen, Basel, Switzerland). Adherent CRFK (ATCC no. CCL-94) and FEA cells were maintained in RPMI 1640 with Glutamax I, while adherent fcwf-4 cells (ATCC no. CRL-2787) were cultured in EMEM (ATCC 30–2003). All media were supplemented with 10% heat-inactivated fetal calf serum (Bioconcept, Allschwil, Switzerland), 100 U/mL penicillin and 100 mg/mL streptomycin (Gibco®, Invitrogen).

ODN 2216 and ODN 2243 were obtained from Alexis biochemicals, Enzo Life Sciences AG, Switzerland for *in vitro* studies and ODN 2216 was synthesized by Microsynth AG, Balgach, Switzerland for the *in vivo* experiment. Recombinant feline IFNα (rfeIFNα) was obtained from PBL Biomedical, Piscataway, New Jersey, USA. All molecules were solubilized in endotoxin-free PBS. ODN 2243 consists of the same sequence as ODN 2216, with CpG motifs inversed to GpC. For *in vitro* experiments, both ODNs and rfeIFNα were diluted in RPMI 1640 with Glutamax I supplemented as described above.

Viability of stimulated cells was compared using the trypan blue exclusion test. Briefly, after stimulation for 24 h with increasing concentrations of ODN 2216, ODN 2243 or equivalent volumes of PBS as control, cells were stained with a 0.4% trypan blue solution (Dr Bender and Dr Hobein AG, Zurich, Switzerland) and percentages of viable cells were compared.

### Proliferation assay

**Figure 1:**
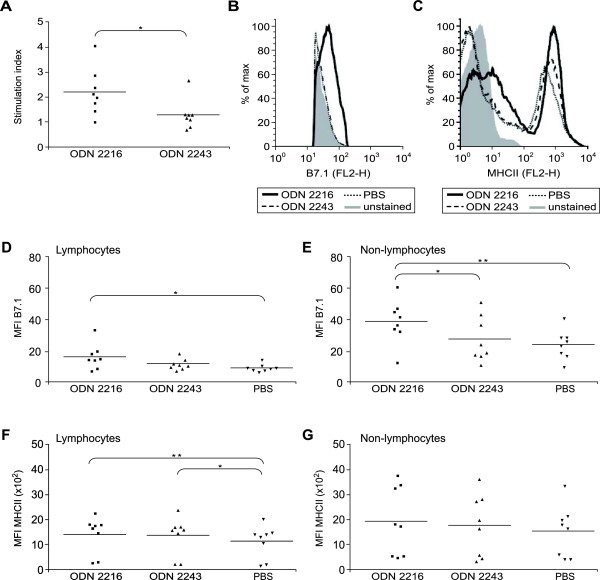
**ODN 2216 induce proliferation of primary feline immune cells and enhance their expression of costimulatory surface molecules.** (**A**) The proliferation of feline PBMCs of eight adult cats belonging to groups 2 and 3 after stimulation with ODN 2216 and ODN 2243 was assessed by H^3^-thymidine incorporation. The dots indicate the stimulation index calculated from triplicate cultures for each cat. (**B** and **C**) Expression levels of B7.1 and MHCII surface molecules were assessed 24 h post stimulation with the indicated treatments by flow cytometry. The fluorescence of gated PBMCs of one cat selected as an example is depicted. Isotype control samples are indicated as unstained. (D-G) Mean fluorescence intensity (MFI) for B7.1 (**D** and **E**) and MHCII (**F** and **G**) of gated lymphocytes and non-lymphocyte subpopulations stimulated with the indicated treatments. Results for eight cats belonging to groups 2 and 3 are shown. *p < 0.05, **p < 0.01.

PBMCs were seeded immediately after isolation at a concentration of 3 × 10^6^ cells/mL in 96-well U-bottom plates. Triplicate cultures for each cat were treated with either 4 μg/mL ODN 2216 or 2243 or an equal volume of endotoxin-free PBS. After an initial incubation of 18 h, the cells were pulsed for 24 h with ^3^ H-thymidine (Perkin Elmer, Schwerzenbach, Switzerland). Standard liquid scintillation protocols were used for harvesting of the cells and uptake of ^3^ H was assessed with the Packard Tri-Carb 1600TR liquid scintillation analyzer (Perkin Elmer). Proliferation rates were calculated as the mean counts per minute (c.p.m) of triplicate cultures. The stimulation index (SI) depicted in Figure 1 was calculated as follows:

$$\frac{\text{Mean c.p.m.of ODN-treated cultures}}{\text{Mean c.p.m of PBS-treated cultures}}$$

### Flow cytometry

PBMCs were treated at a density of 3 × 10^6^ cells/mL with 4 μg/mL ODN 2216 or ODN 2243 or an equivalent volume of endotoxin-free PBS and cultured for 24 h in a 12-well format. During collection of the cells, the adherent cell fraction was removed with 0.05% trypsin-EDTA (Gibco®, Invitrogen). Harvested cells were divided into 3 fractions labeled separately with either anti-feline B7.1 mouse monoclonal IgG (kindly provided by Prof Mary Tompkins, Flow Cytometry and Cell Sorting Laboratory, NC State College of Veterinary Medicine, USA), anti-feline MHCII mouse monoclonal IgG1 (Department of Pathology, Microbiology and Immunology, University of California, Davis, USA) or fluoresceinisothiocyanate (FITC)-conjugated mouse IgG1 as isotype control (BD Bioscience, Allschwil, Swizerland). The fractions were subsequently stained with R-Phycoerythrin (RPE)-conjugated goat anti-mouse IgG1 (BioConcept, Allschwil, Swizerland). Fluorescence data was obtained using the FACSCalibur® instrument (Becton Dickinson, Allschwil, Switzerland) and the CellQuestPro™ software. Gates representing lymphocyte and non-lymphocyte populations were set on the basis of forward versus side scatter, and a total of 50 000 events were acquired in the non-lymphocyte gate. Data was analyzed with the FlowJo software (Tree Star, Olten, Switzerland), whereby an additional gate was set comprising both lymphocyte and non-lymphocyte populations (PBMC gate). MHCII and B7.1 expression levels were determined as mean of fluorescence intensity for each gated cell population. Identical gates were set for all cats in such a way that they comprise the desired cell populations of each animal.

### Relative gene expression analysis

PBMCs were stimulated with ODN 2216, ODN 2243 or endotoxin-free PBS at a density of 3 × 10^6^ cells/mL directly after isolation, while CrFK, FEA or fcwf-4 cells were cultured to confluency prior to stimulation. Experiments were carried out in a 96-well format. For stimulation of adherent cells with supernatants (production see below), total cell culture medium was discarded from the wells and the monolayers were further cultured in 100 μL undiluted supernatants for the rest of the experiment. At time points relevant to each experiment, the supernatants were removed and cells were lysed with mRNA lysis buffer (mRNA isolation kit I, Roche Diagnostics, Rotkreuz, Switzerland). mRNA extractions were performed with the mRNA Isolation Kit I and MagNA Pure LC Instrument (Roche Diagnostics) and first strand cDNA was synthesized with the High Capacity cDNA Reverse Transcription Kit (Applied Biosystems, Rotkreuz, Switzerland). Real-time quantitative PCR (qPCR) reactions consisted of 5 μL cDNA in a total volume of 25 μL per reaction using the TaqMan® Fast Universal PCR Master Mix (Applied Biosystems). Thermocycling conditions included an initial denaturation of 20 s at 95°C followed by 45 cycles of amplification by melting at 95°C for 3 s and annealing at 60°C for 45 s. Primers and probes for feline genes have been previously described [58]. mRNA expression factors of selected genes, which correspond to ratios of mRNA levels measured in ODN 2216 or ODN 2243 stimulated versus PBS stimulated cells, were calculated and normalized with GeNorm version 3.5 [59], using either both feline β-glucuronidase (GUSB) and tryptophan 5-monooxygenase activation protein zeta polypeptide (YWHAZ) (usually) or Glyceraldehyde 3-phosphate dehydrogenase (GAPDH) alone (when specified) as reference genes, under conditions validated for the feline species [60]. Generally depicted in the graphs are mean expression factors calculated from duplicate experiments carried out simultaneously. Where results of one cat are illustrated, experiments were conducted with cells of at least 3 individual animals and representative data is shown.

### Production of supernatants

For each cat, PBMCs were resuspended in supplemented RPMI 1640 with Glutamax I (Gibco®, Invitrogen) at a concentration of 10^6^ cells/mL in 6-well plates and stimulated immediately after isolation with 4 μg/mL ODN 2216, 4 μg/mL ODN 2243 or an equivalent volume of endotoxin-free PBS. After 24 h incubation_,_ supernatants were harvested by centrifugation of the cultures twice at 2000 × *g* for 10 min, aliquoted and stored at −20°C. Large supernatant quantities were produced with PBMCs purified after one blood collection, enabling the utilization of the same supernatants for virus inhibition experiments concerning VSV, FCV, FCoV, FHV and FPV. New supernatant batches were produced for use in FeLV inhibition assays. Supernatants derived from PBMCs stimulated with ODN 2216, ODN 2243 and endotoxin-free PBS are referred to as Sup2216, Sup 2243 and Sup Neg in the text and in the figures.

### Western blot

CrFK and fcwf-4 grown to confluency in 12-well plates were stimulated with 600 μL of PBMC supernatants produced as explained above. At the time points indicated, cells were harvested and counted. 10^6^ cells of either cell line were resuspended in 30 μL sample buffer (0.5 M Tris(hydroxymethyl)aminomethane, 5% SDS, 10% β-mercaptoethanol, 40% glycerol, and 0.05% bromphenol blue) and boiled at 95°C for 5 min. SDS-PAGE separation and submersed immunoblotting procedures were carried out as previously described [61]. The Spectra Multicolor Broad Range Protein Ladder (Fermentas GmbH, St. Leon-Rot, Germany) served as molecular weight standard marker for each blot. For protein visualization, membranes were first cut immediately below the 80kB marker band. The top and bottom membrane fractions were incubated with murine anti-human Mx MAb M143 (generously provided by Dr J. Pavlovic, Institute for Virology, University of Zürich, Switzerland) and murine anti-β-actin monoclonal antibody as a loading control (Sigma Aldrich GMbH, Buchs, Switzerland) respectively. Both fractions were subsequently incubated with a peroxidase-labelled goat anti-mouse IgG (Jackson Immunoresearch, Newmarket, Suffolk, UK). Bands were digitalized using the Chemigenius 2 BioImaging System (Syngene, Cambridge, UK).

### Viruses and viral inhibition assays

VSV Indiana strain (Institute of Virology, Vetsuisse Faculty, University of Zurich, Switzerland), FCoV Wellcome strain (a generous gift from Prof. A. Kipar, University of Liverpool, Great Britain), FPV (kindly provided by Prof. U. Truyen, University of Leipzig, Germany), FHV ZH5-04 strain and FCV F9 strain (kindly provided by Veterinaria AG, Zurich, Switzerland) were titrated on both CrFK and fcwf-4 cells. Viral stock dilutions inducing 95% cytopathic effect (CPE) after 24 h (72 h for FCoV and FPV) were selected for inhibition experiments in order to ensure proper measurement of inhibitory effects. Monolayers of CrFK and fcwf-4 cells in 96-well plates were incubated for 24 h with 100 μL of the supernatants produced with PBMCs from cats of groups 1, 2 and 3. With the exception of assays carried out with FPV, viral inhibition experiments were conducted simultaneously and with supernatants thawed an equal number of times. The treated cells were then inoculated with virus (VSV, FCV, FHV, FCoV) or trypsinized with 0.05% trypsin-EDTA (Gibco®, Invitrogen) and allowed to settle in viral suspension (FPV), and inhibition assays were carried out after 24 h (72 h for FCoV and FPV) according to the procedure described previously [62]. Briefly, supernatants were discarded and cell debris was removed from the wells by 3 cycles of washing with Hank’s balanced salt solution (HBSS) (Gibco, Invitrogen) and shaking on an orbital shaker for 15 s. Remaining cells were fixed with 5% formalin and stained with a crystal violet solution. For spectrophotometric measurements, 100% methanol was added to the dried out wells and absorbance was read at 595 nm on a SpectraMax Plus 384 microtiter plate reader (Molecular Devices, Bucher Biotec AG, Basel, Switzerland). Viral inhibition rates were calculated with the following formula:

$$\frac{\text{Mean optical density}\;\left(\text{OD}\right)\;\text{values of duplicate wells treated with Supernatant}}{\text{Mean OD values of quadruplicate wells treated with medium alone}}$$

FeLV-A Glasgow-1 strain (a generous gift from Prof. M. Hosie and O. Jarret, University of Glasgow, Great Britain) was titrated on FEA cells, and the lowest stock dilution leading to productive infection of the cells after 48 h was used for inhibition assays. Experiments were carried out in 96-well plates and cells were treated with 100 μL of supernatants or relevant controls immediately prior to inoculation. Every second day thereafter, 50 μL culture medium was replaced by the same volume of fresh supernatant. At appropriate time points, cells and supernatants were harvested and total nucleic acid was extracted from both the cells and supernatants using the MagNA Pure LC DNA Isolation Kit I and MagNA Pure LC Instrument (Roche Diagnostics). Viral replication in supernatants and proviral loads in cells were measured by real-time RT-PCR and real-time PCR respectively, with assays previously described [63]. The time course experiments were conducted with supernatants derived from PBMCs of three cats and the measurements on day 8 post inoculation were carried out with material derived from two additional cats. In order to facilitate interpretation of the figures illustrating measurements of viral RNA loads, 45 cycles-cycle threshold (Ct) values were calculated and means of duplicate wells are depicted.

### *In vivo* experiment

Cats c05 and c06 were administered ODN 2216 once subcutaneously at a dose of 0.2 mg/kg body weight while cats c07 and c08 received endotoxin-free PBS as control. The treatments were distributed in equal portions bilaterally in axillary and inguinal regions. Heparinized blood was collected at time points 0 h (just before injection of the treatment), 8 h, 24 h, 48 h, 96 h and 192 h. The cats were clinically examined and body temperature was measured at each time point. Whole blood samples were used in part for the assessment of hematological paramaters with the Sysmex XT 2000iV (Sysmex, Norderstedt, Germany) as previously described [57]. The rest of the blood was mixed with mRNA lysis buffer (Roche Diagnostics) immediately upon blood collection and processed as recommended by the manufacturer. Plasma was obtained by centrifugation of whole blood samples for 10 min at 1500 × *g* and conserved at −80°C until use for *in vitro* experiments. Mx mRNA expression was measured by qPCR and normalized to the expression of two feline housekeeping genes as described above. The expression factors depicted in the figures represent the ratio of mRNA levels from a specific time point to mRNA levels from time point 0 h. For viral inhibition assays, the plasma collected from each cat at each time point was incubated for 24 h with fcwf-4 cells prior to their inoculation with FCV. The assays were carried out as described above and viral inhibition factors were calculated as follows:

$$\frac{\text{Mean optical density}\;\left(\text{OD}\right)\;\text{values of duplicate wells treated with plasma from timepoint}\;0\text{h}}{\text{Mean OD values of duplicate wells treated with plasma from timepoint Xh}}$$

### Statistical analysis

**Figure 2:**
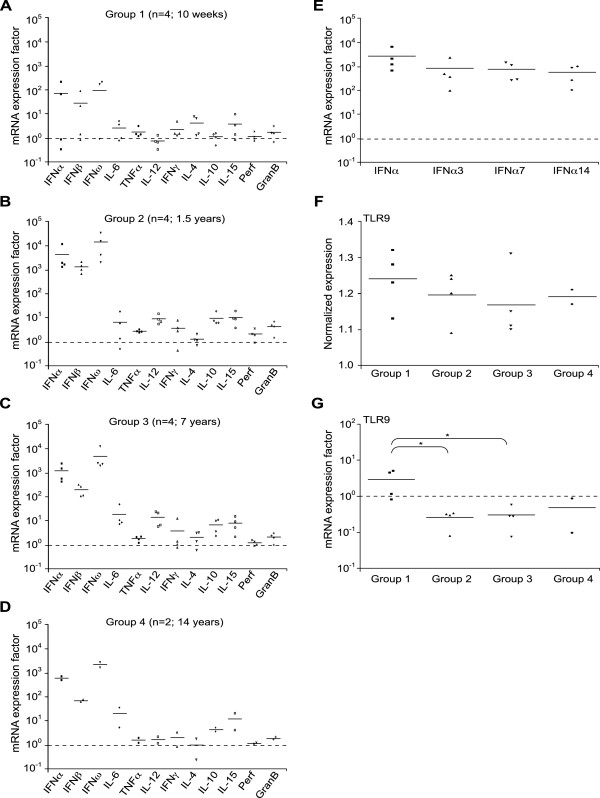
**ODN 2216 induces an antiviral gene expression profile in PBMCs of adult cats.** (**A**-**D**) mRNA expression factors of the indicated genes were measured in PBMCs isolated from fourteen cats belonging to the indicated age groups and stimulated with ODN 2216 for 24 h. (**E**) mRNA expression factors of the indicated IFNα subtype genes were measured in PBMCs isolated from four adult cats (group 2) and stimulated with ODN 2216 for 24 h. (**F**) mRNA levels of TLR9 were measured by real-time qPCR in unstimulated PBMCs of all four groups of cats and normalized to the expression of a feline housekeeping gene (GAPDH). (**G**) Relative TLR9 mRNA expression factors were measured in ODN 2216 stimulated PBMCs from cats of all four groups. *p < 0.05, Perf = perforin, GranB = granzyme B.

All statistical analyzes were performed using GraphPad Prism for Windows, version 3.0 (GraphPad Software, San Diego California USA). Due to the limited number of cats integrated in the study, we refrained from using a parametric approach in the statistical tests. As such tests require a larger sample size than *n* = 4, it was not possible to calculate p-values for the induction in expression of each gene shown in Figure 2. Differences between treatment groups in proliferation of PBMCs were analyzed with a Mann–Whitney test and expression of co-stimulatory molecules on the surface of these cells were analyzed with a Wilcoxon signed rank test, where values for each cat were paired. Relative Mx mRNA expression, OD values from viral inhibition assays, ratios for FeLV provirus and Ct values for FeLV viral RNA loads were analyzed with a Wilcoxon signed rank test with pairing of values for each cat when treatment with different supernatants were compared, or a Mann–Whitney test when incubation with supernatant was opposed to treatment with medium alone or rfeIFNα. Normalized and relative TLR9 mRNA expression ratios between different animal age groups were also compared with a Mann–Whitney test. Longitudinal effects on FeLV viral and proviral loads were compared with each other using a Mann–Whitney test carried out with Area Under the Curve (AUC) values. Correlations were assessed using the Spearman test. p-values < 0.05 were considered statistically significant.

## Results

### Prototype CpG-A, ODN 2216, induce proliferation of primary feline immune cells and enhance their expression of costimulatory surface molecules

Proliferation of PBMCs in response to treatment with CpG ODN gives strong indications about the biological activity of the stimulatory molecule and has been used to screen ODN in many species [25]. In relation to this, the potential of CpG-A to induce proliferation of feline PBMCs were assessed by measurement of ^3^ H-thymidine incorporation after stimulation. Although considerable variability was observed between individuals, a 2-fold increase in proliferation was observed after stimulation with ODN 2216 when compared to stimulation with inactive control ODN 2243 (*p* = 0.0281, Figure 1A). The cells of three cats (c08, c09, c12) could be stimulated to particularly high proliferative rates by ODN 2216 (values above the mean of eight cats illustrated in Figure 1A) in the following order: c08 > c12 > c09.

Another characteristic feature of stimulatory ODN is their ability to enhance interactions between various immune cell populations by upregulation of cell surface costimulatory molecules. With the objective to test whether ODN 2216 could exert such properties in feline immune cells, the expression of B7.1 and MHCII was measured by flow cytometry in stimulated PBMCs of the same eight cats as above. The expression of both co-stimulatory molecules was evaluated in gates defined to contain a PBMC population, a lymphocyte population and a non-lymphocyte population of cells. The observed effects varied considerably between the cells of individual cats, ranging from no alterations to an increase of 400% stained cells in some cellular subpopulations after ODN 2216 stimulation. Also, in some animals, stimulation with the control ODN 2243 indicated similar staining patterns as PBS, whereas in other cats the induction of effects comparable to those of ODN 2216 could be observed. The response of cells originating from a particular cat to stimulation with ODN 2243 did not however correlate with the influence of ODN 2216 on the expression of either surface molecule on these same cells. Overall, an increased expression of both B7.1 and MHCII was measured in gated PBMCs upon a 24 h stimulation with ODN 2216 when overlaid with the expression of these molecules after ODN 2243 or PBS stimulation (Figure 1B and C). Considering all eight cats, lymphocytic cells expressed significantly higher levels of B7.1 and MHCII when exposed to ODN 2216 than after treatment with PBS (*p* = 0.0195 and *p* = 0.039 respectively, Figure 1D and F). Feline MHCII expression on lymphocytes also seemed to be affected by ODN 2216 and ODN 2243 in a similar manner (Figure 1F). For non-lymphocytic cells, ODN 2216 more specifically affected the expression of B7.1 molecules, inducing significant upregulation of surface levels of this molecule when compared to stimulation with ODN 2243 (*p* = 0.0391) and PBS (*p* = 0.0039) for 24 h (Figure 1E). In contrast, the expression of MHCII was not significantly altered in cells gated as non-lymphocytes at this time point after stimulation (Figure 1G). Finally, the cells of the three cats (c08, c09 and c12) that exhibited the highest proliferation rates in response to ODN 2216 (Figure 1A) also indicated the strongest increase in expression of both cell surface molecules in all cellular subpopulations tested.

### ODN 2216 influence type I IFN and proinflammatory gene expression in primary feline immune cells

**Figure 3:**
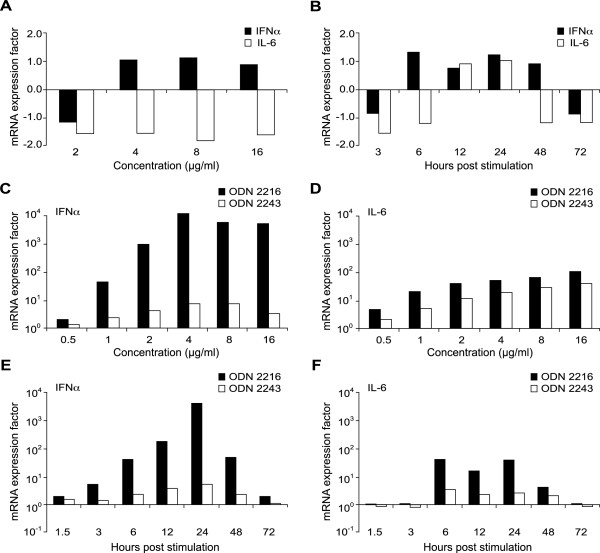
**ODN 2216 influence IFNα and IL-6 expression in primary feline immune cells.** mRNA expression factors of IFNα and IL-6 were measured in (**A**, **B**) fcwf-4 cells stimulated with ODN 2216 and (**C**-**F**) feline PBMCs from one cat belonging to group 2 stimulated with either ODN 2216 or control ODN 2243. The transcription of both genes was assessed either 24 h after treatment of the cells with increasing concentrations of ODN (**A, C, D**), or over time after a single stimulation with 4 μg/mL ODN (**B, E, F**). Experiments **C-F** were repeated for 4 adult cats and representative results are shown.

Through interaction with the TLR9, CpG-A typically induce expression of both type I IFN and proinflammatory cytokines in stimulated cells [12]. In order to understand whether ODN 2216 exert similar effects in the cat, treated cultures of feline PBMCs, FEA, CrFK and fcwf-4 cells were systematically screened for increased mRNA expression of IFNα and IL-6 following stimulation. Although all tested immortalized feline cell lines expressed TLR9 mRNA (data not shown), they failed to respond to stimulation with ODN 2216 (Figure 3A and B and data not shown). However, this molecule exhibited potent immunomodulatory properties in feline PBMCs: when measured 24 h post stimulation, a concentration of only 1 μg/mL ODN 2216 was sufficient to enhance transcription of IFNα by 40-fold, and a maximum induction of this gene was observed when 4 μg/mL ODN were used (Figure 3C). Although affected in a similar pattern, the mRNA expression of the proinflammatory cytokine IL-6 remained comparatively low at all concentrations tested (Figure 3D). In experiments foreseen to determine gene expression kinetics in feline cells after stimulation with the ODN 2216 molecule, an influence on IFNα transcription could be measured as early as 3 h after treatment of PBMCs, whereas increased levels of IL-6 mRNA were observed only as of 6 h post stimulation (Figure 3E and F). Notably, the highest induction of both genes was measured 24 h after addition of ODN 2216 to the cultures, with transcription increasing by 9000-fold and 39-fold at this time point for IFNα and IL-6 respectively (Figure 3E and F). The observed effects were specifically conferred by the CpG motifs comprised in the 2216 ODN, since specific control ODN 2243 only induced slight elevations in the expression of the genes tested (Figure 3C-F). Importantly, trypan blue exclusion experiments indicated no evidence of cellular toxicity after treatment neither with ODN 2216 nor with ODN 2243 at all concentrations tested (data not shown).

### ODN 2216 broadly influence the gene expression profile in primary feline immune cells of adult cats

In an effort to assess both the breadth of the effects conferred by treatment with a CpG-A and possible variability in the responses obtained in individual cats, the mRNA expression of ten genes relevant to early immune responses was measured in ODN 2216 stimulated PBMCs of fourteen cats divided in four different age groups (group 1: 10 weeks (*n* = 4), group 2: 1.5 years (*n* = 4), group 3: 7 years (*n* = 4), group 4: 14 years (*n* = 2)). Only slight individual variability in gene induction was observed after 24 h stimulation of immune cells from adult cats ranging between 1.5 and 14 years of age (Figure 2B-D). Overall, the mRNA expression profile measured in stimulated PBMCs of these cats corroborated their induction of strong antiviral immune responses. The expression of type I IFN mRNA, including IFNα, IFNβ and IFNω was substantially increased in the immune cells subjected to ODN 2216 stimulation from every adult cat, with minimal inductions of 490, 60 and 1600-fold respectively observed in the older animals of group 4 (Figure 2D). Moreover, all individual IFNα subtypes tested were induced at similar levels in the cells of four adult cats from group 2 (Figure 2E). Increased levels of proinflammatory cytokine mRNA were also measured in most individuals of groups 2–4, with IL-6 more systematically increased than TNFα. The cells from these cats indicated a typical Th1 orientation after stimulation, with enhanced transcription of IL-12 in 9/10 and IFNγ in 6/10 animals, together with absent or only low induction of IL-4. Stimulation of PBMCs with ODN 2216 also created an optimal environment for NK cell activity, as indicated by the increases in mRNA expression of the NK cell stimulator IL-15 by up to 20-fold and of the NK cell effector Granzyme B by up to 7-fold. The cells of those cats (c08, c09, c12) that had most effectively proliferated (Figure 1A) and/or exhibited the strongest expression of co-stimulatory molecules (Figure 1B-E) following ODN 2216 stimulation also consistently expressed the highest mRNA levels of IFNα, IFNω, IL-6, Il-12, IL-15 and Granzyme B. PBMCs of cat c08 (group 2, Figure 1B) were also by far most responsive to stimulation with ODN 2216. When cells from kittens of group 1 were stimulated, higher individual variability was observed than in adult animals (Figure 2A). The PBMCs of only 2 out of 4 cats from this group could be stimulated with ODN 2216 to increase mRNA expression of the tested genes. In both individuals, not only the mRNA expression of type I IFN genes was enhanced at much lower levels than observed in adult cats but the overall immune response favored a Th2 direction, characterized by upregulated IL-4 and downregulated IL-12 mRNA levels (Figure 2A).

In order to determine whether a discrepancy in expression of TLR9 between the PBMCs of adult animals and kittens could play a role in these observations, mRNA levels of this gene were measured in immune cells of each cat. Although basal TLR9 expression was similar in the PBMCs of the cats of all age groups (Figure 2F), ODN 2216 stimulation increased TLR9 transcription in the cells of kittens, but decreased transcription of this gene in the cells of adult cats so that differences in the mRNA levels of this receptor were significantly higher in young cats (group 1) than in adult cats of groups 2 and 3 (*p* = 0.0286) (Figure 2G).

### ODN 2216 induce the production of soluble molecules that activate intracellular antiviral mechanisms in feline target cells

**Figure 4:**
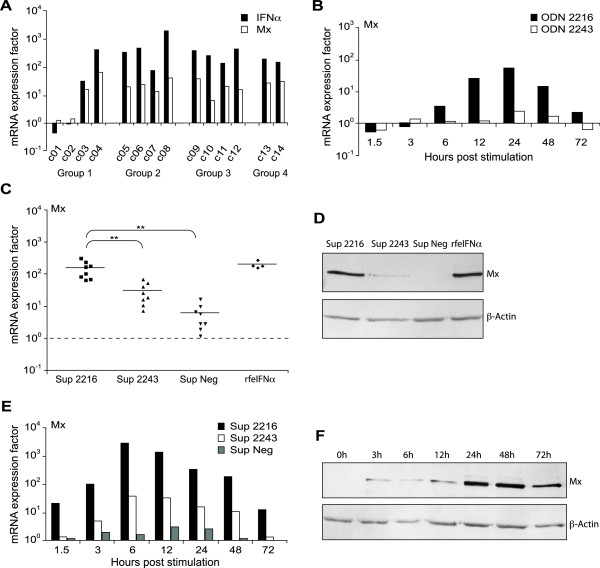
**ODN 2216 induces an antiviral state both in stimulated PBMCs directly and in target cells incubated with supernatants from stimulated PBMCs.** (**A**) mRNA expression factors of the indicated genes were measured in PBMCs of the individual cats (c01-c14) from four different age groups after stimulation with ODN 2216 for 24 h. (**B**) Mx mRNA expression factors were assessed at the indicated time points in PBMCs of one cat after a single stimulation with either ODN 2216 or ODN 2243. (**C**) Mx mRNA expression factors were measured in fcwf-4 cells incubated for 24 h with supernatants (Sup 2216, Sup 2243, Sup Neg) derived from PBMCs of eight adult cats (groups 2 and 3) or 100U recombinant feline IFNα (rfeIFNα). (**D**) Mx protein was detected by Western blot in fcwf-4 cells incubated with the indicated supernatants derived from PBMCs of one cat belonging to group 2 or with 100U recombinant feline IFNα (rfeIFNα) for 24 h. (**E**) Mx mRNA expression factors were measured in fcwf-4 cells at the indicated time points after a single stimulation with Sup 2216, Sup 2243 and Sup Neg respectively. (**F**) Mx protein was detected by Western blot in fcwf-4 cells at indicated time points after stimulation with Sup 2216 derived from PBMCs of the same cat (right panel). **p < 0.01. rfeIFNα = recombinant feline IFNα.

Protective properties of type I IFN against viruses originate from their ability to trigger the production of potent antiviral proteins in nearby cells. The expression of one of these antiviral proteins, the Mx GTPase, is known to be directly stimulated by type I IFN and can be used as marker for the induction of intracellular antiviral mechanisms by these cytokines in cats as well as in other species [19,64]. Thus, as detection of feline type I IFN on a protein level is rendered difficult by the unavailability of antibodies specifically recognizing these proteins, mRNA levels of Mx were measured in feline PBMCs after stimulation with ODN 2216, as indication for production of type I IFN. Transcription of Mx was significantly enhanced and remained proportional to mRNA expression of type I IFN in stimulated PBMCs of individual cats (Figure 4A). Moreover, peak levels of Mx mRNA were measured 24 h after stimulation of the cells, indicating the presence of optimal effects of the type I IFN present in the cell culture medium at this time point (Figure 4B). In order to further determine the potential of the type I IFN liberated by ODN 2216 stimulated PBMCs to induce intracellular antiviral mechanisms in non-immune target cells, cell-free supernatants of PBMCs derived from the blood of individual adult cats and stimulated *in vitro* for 24 h with ODN 2216, ODN 2243 or endotoxin-free PBS (Sup 2216, Sup 2243 and Sup Neg respectively), were incubated with CrFK and fcwf-4 cells. Mx gene transcription was significantly enhanced in cells treated with Sup 2216 compared to Sup 2243 (*p* = 0.0078) and Sup Neg (*p* = 0.0078), at levels comparable to those achieved by stimulation of the cells with 100U recombinant feline IFNα (rfeIFNα) (Figure 4C and data not shown). The Sup 2216 derived from cells of individual animals (c08, c09, c12) that had shown particularly strong responses to ODN 2216 in previous experiments also induced the highest Mx mRNA expression in both cell lines tested. Although treatment with Sup 2243 systematically increased Mx transcription in target cells, protein levels remained low (Figure 4D and data not shown). Furthermore, peak induction of Mx transcription was observed in both cell lines already within 6 hours of incubation with Sup 2216 (Figure 4E and data not shown), while protein levels achieved maximum levels within 24 h post stimulation and remained stable thereafter for at least another 24–48 h (Figure 4F and data not shown).

### ODN 2216 inhibit replication of common feline viruses *in vitro*

**Figure 5:**
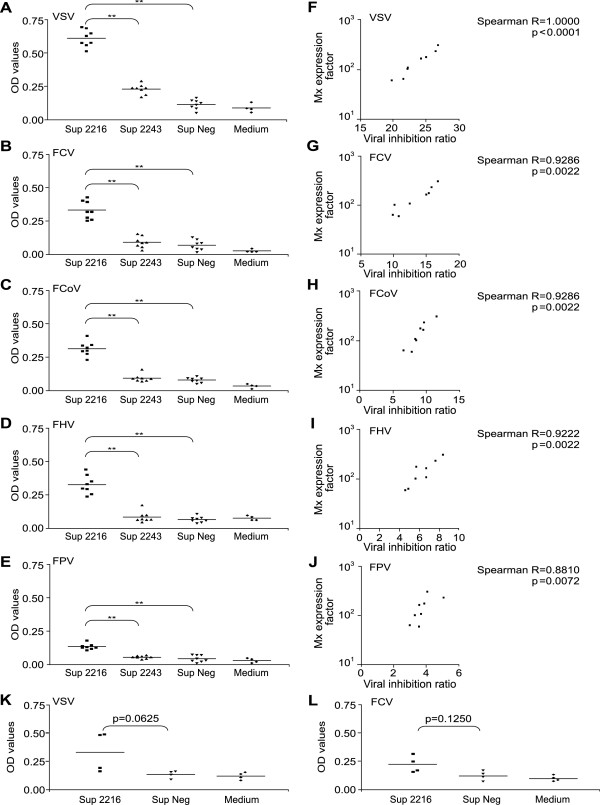
**Supernatants derived from ODN 2216 stimulated PBMCs inhibit viral replication in target cells.** (**A**-**E**) fcwf-4 cells were incubated for 24 h with the indicated supernatants derived from PBMCs of eight adult cats (groups 2 and 3) or medium only as control before inoculation with the indicated viruses. Each dot represents mean optical density (OD) values from spectrophotometric readings of viral inhibition assays conducted on duplicate wells treated with supernatants from an individual cat. (**F**-**J**) Correlation of individual inhibition ratios of each virus with Mx mRNA expression induced in fcwf-4 cells incubated with supernatants of ODN 2216 stimulated PBMCs from the eight cats of groups 2 and 3. Note the different scale on the x-axis for each graph indicating the differences in the inhibitory effects of these supernatants on the different viruses. (**K**, **L**) fcwf-4 cells were incubated for 24 h with the indicated supernatants derived from PBMCs of 4 kittens (group 1) or medium only as control before inoculation with VSV or FCV. Each dot represents mean OD values from spectrophotometric readings of viral inhibition assays conducted on duplicate wells treated with supernatants from one cat. **p < 0.01. VSV = vesicular stomatitis virus, FCV = feline calicivirus, FPV = feline parvovirus, FCoV = feline coronavirus, FHV = feline herpes virus.

Felids are frequently affected by four viruses of different families: the feline herpesvirus (FHV), calicivirus (FCV), parvovirus (FPV) and coronavirus (FCoV). Although these viruses cannot productively infect purified PBMCs *in vitro*, they share the ability to induce cytopathic effects (CPE) in CrFK and fcwf-4 cells. These feline cell lines, however, do not alter their mRNA levels of genes selected as markers for innate immunity upon direct treatment with ODN 2216 (Figure 3A and B and data not shown), preventing from assessing the potential of this molecule to inhibit viral replication directly in these cells. Thus, CrFK and fcwf-4 cells were incubated, prior to their inoculation, with the cell-free supernatants of PBMCs mentioned above: Sup 2216, Sup 2243 and Sup Neg. To this aim, supernatants derived from PBMCs treated for 24 h were selected, as Sup 2216 produced by the cells of several adult cats were estimated to contain optimal type I IFN amounts at this time point, according to the induction of Mx transcription measured directly in these immune cells (Figure 4A and 4B). CrFK and fcwf-4 target cells were then incubated with the supernatants for 24 h before inoculation, as this time span had indicated highest induction of antiviral mechanisms (Figure 4F). The antiviral effects of the supernatants were initially tested on vesicular stomatitis virus (VSV) as control, as this virus is widely recognized for both its potential to induce CPE in cell lines of multiple species and for its particularly high sensitivity to the effects of type I IFN [65,66]. When the Sup 2216 derived from the PBMCs of eight adult cats that had broadly responded to *in vitro* ODN 2216 stimulation (Figure 2B and C, groups 2 and 3) were incubated with fcwf-4 cells prior to their inoculation, significant inhibition of VSV replication was observed (*p* = 0.0039) (Figure 5A). The replication of this virus was also to some degree repressed by Sup 2243 (*p* = 0.0078), an observation reminiscent of the slight induction of Mx in target cells incubated with these supernatants (Figure 4C and D). In turn, the propagation of FCV, FCoV, FHV and FPV on fcwf-4 cells was also significantly suppressed by the Sup 2216 when compared to Sup 2243 (*p* = 0.0039, *p* = 0.0039, *p* = 0.0078 and *p* = 0.0039 respectively) and Sup Neg (*p* = 0.0039), however with expected lower sensitivity than VSV (Figure 5B-E and Table 1). Both Sup 2243 and Sup Neg failed to inhibit replication of this heterogeneous group of feline viruses, underlining the essential role of the 2216 molecule in conferring the observed effects. Importantly, cells stimulated with ODN 2216 directly did not indicate any resistance to viral replication, in concordance to their impaired response to this molecule already measured on a genetic level (Figure 3A and B and data not shown). With respect to the younger cats, the Sup 2216 of those 2 kittens whose cells responded to ODN 2216 stimulation (Figure 2A, group 1) could also inhibit both VSV and FCV on fcwf-4 cells, while the supernatants derived from the PBMCs of the other 2 kittens indicated no inhibition potential on these viruses (Figure 5K and L). Altogether, the viral suppression potential of Sup 2216 from all the cats could be compared to that conferred by treatment of the cells with 10 to 100U rfeIFNα, a quantity determined in titration experiments of rfeIFNα conducted together with the viral inhibition assays (data not shown).

**Table 1:** Means of viral inhibition rates measured in fcwf-4 cells after treatment with supernatants derived from stimulated PBMCs of 8 adult cats

	**VSV**	**FCV**	**FCoV**	**FHV**	**FPV**
Sup 2216	23.49	12.94	8.85	6.18	3.74
Sup 2243	8.74	3.53	2.68	1.65	1.20
Sup Neg	4.26	2.65	2.25	1.29	1.52

Depicted are mean viral inhibition rates for 8 cats. Viral inhibition rates for each cat were calculated with the following formula:

$\frac{\text{Mean optical density}\;\left(\text{OD}\right)\;\text{values of duplicate wells treated with Supernatant}}{\text{Mean OD values of quadruplicate wells treated with medium alone}}$.

*Abbreviations: VSV* Vesicular Stomatitis Virus, *FCV* Feline Calicivirus, *FCoV* Feline Coronavirus, *FHV* Feline Herpesvirus, *FPV* Feline Parvovirus.

The differential inhibition of the viruses tested by the same supernatants is reflected by the distinct viral inhibition ratios observed (Figure 5B-E and Table 1). Sensitivity of each virus to rfeIFNα correlated with sensitivity to the Sup 2216 and induction of Mx in target cells by the supernatants highly correlated with the inhibition of all viruses (Figure 5F-I). Finally, Sup 2216 derived from PBMCs of cats c08, whose cells had indicated strong responsiveness to stimulation with ODN 2216 in previous experiments, most efficiently inhibited the replication of all viruses.

Similar results were notably obtained when the supernatants of PBMCs derived from all cats were incubated with CrFK cells prior to their inoculation with all the above-mentioned viruses (data not shown). This cell line has previously indicated less sensitivity to the antiviral effects of type I IFN [66] and generally 10-fold higher amounts of rfeIFNα were found in titration experiments to be required for the inhibition of all five viruses. Concordantly, average fold viral inhibition in CrFK cells by the Sup 2216 was approximately half that observed in fcwf-4 cells.

### ODN 2216 inhibits replication of a retrovirus *in vitro*

**Figure 6:**
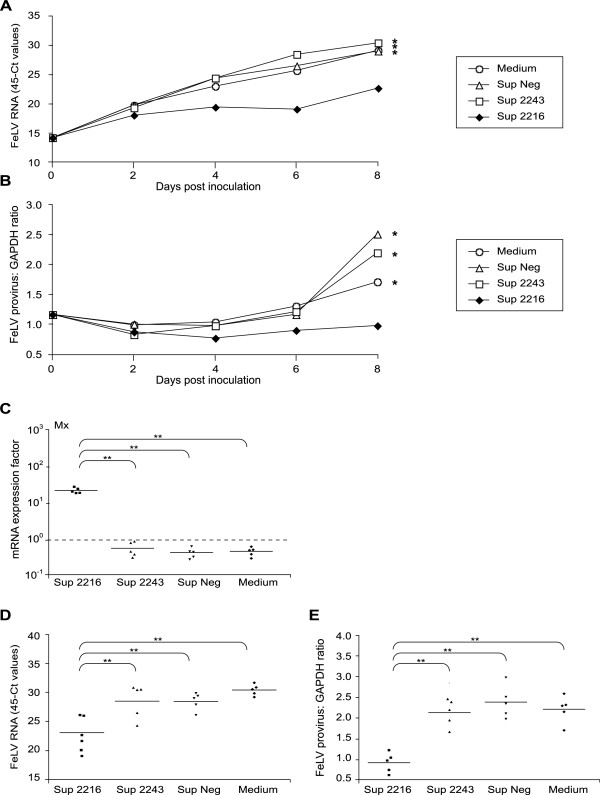
**Supernatants derived from ODN 2216-stimulated PBMCs decrease retroviral DNA and RNA loads in target cells.** (**A**) FEA cells were incubated for 24 h with the respective supernatants or medium alone, before inoculation with the feline leukaemia virus (FeLV), as well as every 2 days thereafter. Viral RNA loads were measured at the indicated time points by real time RT-PCR and 45-Ct values are depicted. (**B**) FeLV DNA loads in the cells were measured at the indicated time points and Ct values were normalized to detection of a housekeeping gene (GAPDH). Mean values from duplicate experiments carried out simultaneously with the supernatants derived from PBMCs of a selected cat (belonging to group 2) and with medium alone are shown as an example. Results for (**A**) and (**B**) are indicative of those obtained with supernatants from PBMCs of two additional adult cats (from group 3). Stars represent statistical differences in area under the curve (AUC) measurements between the curves of all three cats obtained in cells incubated with Sup 2216 and each of the other treatments. Mx mRNA expression factors (**C**), viral loads (45-Ct values depicted) (**D**) and proviral loads (**E**) were measured in the FEA cells of five cats (two from group 2 and three from group 3) on day 8 post inoculation. Each dot represents the mean of duplicate measurements for an individual cat. *p < 0.05, **p < 0.01.

The life cycle of retroviruses is characterized by the reverse transcription of their genomic RNA into DNA and subsequent integration of this viral DNA as provirus into the genome of the host, causing permanent infection most often accompanied by persistent virus production by infected cells. The feline leukemia virus (FeLV), a gammaretrovirus that can be propagated on FEA cells *in vitro*, affects domestic cats worldwide. As viral replication in chronically infected cats can be lowered by treatment with IFNα [67], the potential of Sup 2216 produced by the PBMCs of five adult cats (c06 and c08 from group 2; c09, c10, c12 from group 3) to inhibit productive infection of FEA cells was analyzed. In initial experiments, this cell line exhibited similar responses as fcwf-4 and CrFK cells to both direct treatment with ODN 2216 and incubation with the different supernatants (Figure 3A, 4C and D and data not shown). Compared to incubation with medium alone, treatment of FEA cells with Sup 2216 for 24 h prior to inoculation with FeLV followed by repetitive treatments of the cells with this supernatant every 2 days thereafter significantly reduced viral RNA (*p* < 0.05) and DNA (*p* < 0.05) measured in the cell culture supernatants and cells respectively as of 4 days post inoculation (Figure 6A and B). The antiviral potential of the supernatants from the individual cats was very similar; however the best results were conferred by the Sup 2216 derived from cells of c08 (depicted in Figure 6A and B).The kinetic curve of viral RNA loads in cultures of cells treated with Sup 2216 of this cat was similar to those obtained in cells treated with 50U rfeIFNα (data not shown). Also, treatment of the cells with ODN 2216 directly affected neither viral RNA nor viral DNA loads measured in the supernatants and the cells respectively (data not shown). Mx mRNA expression was 80-fold higher in the Sup 2216 treated cells than in all controls 8 days post inoculation, indicating the ability of these supernatants to sustain antiviral mechanisms when applied to the cells repeatedly (Figure 6C). Furthermore at this time point, the Sup 2216 treated cells exhibited significantly lower viral DNA loads (*p* = 0.0313) and produced significantly less virus (*p* = 0.0313) than cells treated with Sup 2243, Sup Neg or medium alone (Figure 6D and E). The extent of Mx transcription conferred by the Sup 2216 of the cats strongly correlated with lower provirus (*p* = 0.0053) and virus (*p* = 0.0012) loads measured in the FEA cells and supernatants respectively on day 8 post inoculation (*p* = 0.0053). Also, the highest Mx mRNA levels in target cells were conferred by Sup 2216 of cat c08 and reflected by the lowest viral and proviral loads measured at this time point in our experiments.

### ODN 2216 induces an antiviral state in the domestic cat *in vivo*

**Figure 7:**
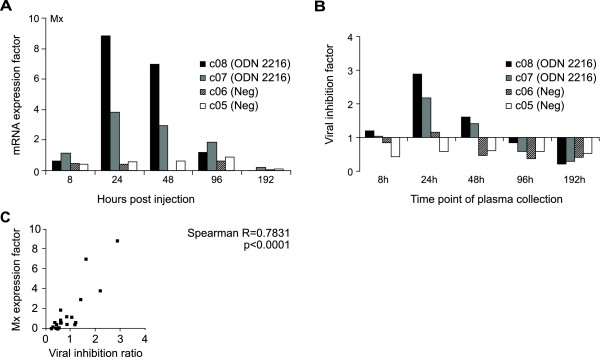
**Subcutaneous injection of ODN 2216 induces a systemic antiviral state *in vivo.*** (**A**) The cats received an injection of either 200 μg/kg ODN 2216 (cats 1 and 2) or endotoxin-free PBS (cats 3 and 4). Mx mRNA expression was measured by qPCR in whole blood. Depicted values represent the ratio of mRNA levels for a given cat (c05-c08) at the indicated time point to mRNA levels for the same cat at time point 0 h. (**B**) fcwf-4 cells were incubated for 24 h in duplicates with plasma collected from the cats 1 to 4 at the indicated time points post injection, and inoculated with the FCV. Indicated are viral inhibition factors calculated as described in the material and methods section. (**C**) Mx mRNA expression factors measured in the blood of all four cats at time points 8 h-192 h were correlated with the inhibition of FCV induced *in vitro* by the corresponding plasma samples.

In a final step, the induction of antiviral responses by ODN 2216 was measured *in vivo* and characterized using *ex vivo* assays. The molecule was administered once subcutaneously to two cats (c07 and c08), while two other cats (c05 and c06) received endotoxin-free PBS as a negative control. The injections were well tolerated by all cats: no rise in body temperature, no local reactions at the injection site and no other undesired side effects were noted. Absolute monocyte counts increased 1.5 to 2-fold in the first 12 h in cats that had received ODN 2216 (data not shown). No other treatment-specific alterations were noted in hematological values throughout the experiment. Mx expression was measured in blood at early time points after injection as marker for the induction of antiviral immune processes. mRNA levels of Mx increased by 8.8 and 3.8-fold within 24 h in both treated cats and decreased gradually thereafter until 192 h post treatment (Figure 7A). In accordance with observations from *in vitro* experiments, cat c08 exhibited a stronger response to ODN 2216 than cat c07. No increase in Mx expression was observed in the blood of the control cats (c05 and c06). In a further experiment, plasma was collected from all four cats at regular intervals post injection and was utilized in an *in vitro* inhibition assay. The plasma obtained from both treated cats 24 and 48 h after administration of the molecule was able to inhibit replication of FCV *in vitro* (Figure 7B). In contrast, plasma from untreated cats seemed to slightly enhance the susceptibility of target cells to FCV inoculation.

## Discussion

In the present study, we conducted both *in vitro* and *in vivo* experiments that characterize immunomodulatory and antiviral effects of a CpG-A molecule in the domestic cat. We show that ODN 2216, the first described CpG-A [12], can upregulate the expression of a series of genes in feline immune cells that play important roles in early responses to viruses. Soluble molecules produced by feline PBMCs upon ODN 2216 stimulation significantly increased resistance of various feline target cell lines to propagation of viruses from five different families, namely FCV, FPV, FHV, FCoV and FeLV. The observed repression of viral replication highly correlated with the mRNA expression of type I IFN genes in stimulated PBMCs as well as the induction, prior to inoculation, of antiviral mechanisms in the target cells. Furthermore, a single administration of ODN 2216 significantly increased the expression of Mx in the blood of treated cats. Plasma from these animals could also inhibit replication of FCV *in vitro*. Our data underline the prophylactic potential of ODN 2216 in an outbred species as a stand-alone agent and against a large range of viral pathogens simultaneously. Cats may highly benefit from such a molecule when placed in environments with strong infectious pressure such as catteries, shelters or pet shows.

Cells of the feline immune system were strongly influenced when cultured in the presence of ODN 2216. First, feline PBMCs significantly proliferated in presence of ODN 2216, indicating a direct and /or indirect stimulation of one or more immune cell subpopulations by this molecule. Although in contrast to other classes of ODN CpG-A does not generally induce lymphocyte proliferation in mice [12], similar effects have previously been observed in ovine cells, where higher concentrations of CpG-A could induce minimal levels of proliferation [68]. Additionally, ODN 2216 increased the expression of cell surface co-stimulatory molecules in PBMCs, reinforcing possible interactions between various immune cell populations in subsequent specific responses. While B7.1 molecules were upregulated on both lymphocytic and non-lymphocytic cells after ODN 2216 stimulation, MHCII expression could only be increased on lymphocytic cells. This observation may be linked to the time point of our measurements, or to the necessity of transport to the cell surface of MHCII molecules coupled with antigen for presentation to other immune cells. Finally, the transcription of a series of markers of innate immune responses was considerably influenced in feline PBMCs by stimulation with ODN 2216. The most astonishing effects of this molecule thereby remain the potent induction of IFNα and IFNω, with mRNA expression of these genes increased by up to 12 000 and 35 000-fold respectively in PBMCs of certain animals. In line with observations published recently, the induction of IFNγ by this CpG ODN remained moderate [34]; nevertheless, the measurements carried out 24 h after stimulation of purified feline cells support induction of Th1-oriented immune responses and enhanced NK cell activity, both highly desired in the contexts of viral infection.

Variability in the immunomodulatory and antiviral effects of ODN 2216 on feline cells was mainly related to the age of the cats. Among middle-aged adult cats, consistency in the stimulatory potential of this molecule was reflected through the highly similar patterns in gene expression profiles induced in immune cells after stimulation, as well as through the moderate deviations in those experiments aiming to characterize the immunomodulatory properties of ODN 2216 (Figure 1). The cells of several animals however, in particular cat c08, but also c09 and c12, indicated particularly strong responsiveness to stimulation throughout the study. Such observations are most likely linked to the genetic variability of individuals of an outbred species, as inherited factors are known to play an important role in the magnitude of innate immune responses [69]. With respect to this observation, it should be noted that the SPF origin and maintenance in a barrier facility of the animals included in this study may slightly lower the variability in strength and breadth of innate immune responses, and studies with cells obtained from field cats would give further information on divergence in response to stimulation of the innate immune cells of individual animals. The immune cells of cats of 14 years of age seemed to respond slightly less well to ODN 2216 than those of younger adult animals, while PBMCs of kittens indicated much more reticence to stimulation, with either limited or absent upregulation of both type I IFN and other genes measured after incubation with ODN 2216. Stimulated PBMCs from this group of young animals moreover indicated a tendency to develop an immunologic environment with a Th2 orientation, including higher production of IL-4 and impaired induction of IL-12 compared to cells from adult cats. Although the kittens included in this study were already of 10 weeks of age, these observations strongly corroborate the immature IFN system of neonatal mice [70], the impaired immune cell activation via TLR9 in human neonatal mononuclear cells [71,72] and the bias towards Th2 rather than Th1 responses in human fetuses and neonates [73,74]. Furthermore, concordantly to findings in human neonatal blood [75,76], basal TLR9 transcription in kittens indicated levels similar to those of adults. However, ODN 2216 stimulation increased mRNA expression of this gene only in the young animals (Figure 2C). These results open new perspectives on possible explanations for the qualitative discrepancy between innate immune responses in newborns and adults. The availability of specific antibodies to feline TLR9 would greatly support further investigations in this direction.

The experiments conducted both *in vitro* and *in vivo* in this study underline the feasibility of utilizing Mx as a marker for induction of resistance to viral infections in the domestic cat. Admittedly, both the supernatants of PBMCs stimulated with CpG-A and serum of treated cats contain a mixture of molecules that could play a role in the observed antiviral effects. However, the viral inhibition in our *in vitro* assays was similar to that obtained after treatment of the cells with rfeIFNα and highly correlated with the induction of Mx transcription in the cell lines incubated with the supernatants. After ODN 2216 injection *in vivo*, Mx expression in plasma also strongly correlated with its antiviral potential. It is however important to note that Mx mRNA and protein cannot be mechanistically linked to inhibition of the viruses tested. Although the expression of other antiviral molecules such as 2'5'oligoadenylate synthetase (OAS) and the RNA-dependent protein kinase (PKR) was not measured, their induction has been reported following CpG ODN stimulation of PBMCs in other species [77,78] and differential interplay between several effector mechanisms most likely leads to the inhibition of individual viruses.

In comparison to ODN of other groups, CpG-A have been used in much fewer studies concerning innate antiviral responses. This is most likely the result of the more time consuming and costly manufacturing process of these molecules linked to the synthesis of the flanking poly (G) strings necessary for optimal immune effects [31]. Also, CpG-A exhibit only weak stimulation of B cells, which produce antibodies that are valuable in later antiviral responses. Stability of CpG-A *in vivo* has also been questioned due to the only partial phosphorothioate backbone of these molecules. This can be partially compensated however, by the formation, through association of their poly (G) stretches, of highly ordered G-tetrad structures with enhanced stability [12]. Their smaller content in synthetic backbones could moreover help reduce possible long-term side effects [79,80]. Most importantly, CpG-A remain the most potent inducers of type I IFN, themselves the most biologically active antiviral molecules known to date [19]. With regard to this, a recombinant feline type I IFN marketed in both Japan (Intercat®) and Europe (Virbagen Omega®) has made its way into therapeutic protocols for FCV, FHV, FeLV and canine parvovirus infections [67,81-86] and has demonstrated preventive capacities in a cattery developing an outbreak of FPV [87]. However, when compared to direct initiation of antiviral mechanisms by a recombinant IFNα protein, administration of CpG ODN holds the advantage of inducing the production of all type I IFN and their subtypes, which have been shown to possess differential antiviral properties and kinetics [88]. Our data demonstrate that five viral species belonging to the Calicivirus, Herpesvirus, Parvovirus, Coronavirus and Retrovirus families were highly sensitive to the immunologic effects of ODN 2216 in feline cells. Strength and breadth of antiviral defense mechanisms induced *in vivo* by this molecule have the potential to outweigh those observed with recombinant IFN.

In frame with the results of our *in vitro* experiments, the administration of ODN 2216 *in vivo* could promote an antiviral state in the domestic cat in the absence of adverse reactions. Just as stimulated PBMCs exhibited high mRNA expression of type I IFN and only a marginal increase in mRNA levels of the proinflammatory cytokines IL-6 and TNFα, treated cats showed significantly increased Mx expression in their blood and lacked flu-like symptoms generally linked with administration of immunostimulatory molecules *in vivo*. Additionally, although our *in vivo* studies included a limited number of cats, the strength of responses to ODN 2216 stimulation *in vitro* and *in vivo* for individual animals strongly correlated, indicating that individual sensitivity to stimulation most likely can be predicted with *in vitro* experiments. Our data further show that a single subcutaneous injection of ODN 2216 sufficed to induce the systemic presence of antiviral molecules for 24 to 36 h in the plasma of treated cats; during this time, an increase of Mx expression of at least 4-fold in the blood of treated cats was indicative of significant inhibition of viral replication *in vitro*. These observations support possible resistance to viral infection by ODN 2216 for several days, a phenomenon described already in mouse models [89].

Altogether, this study underlines the strong potential for ODN 2216 in the prevention of a large variety of viral diseases. CpG-A molecules should thus not be left aside when stimulating innate immunity for prophylactic purposes. Future studies should aim at optimizing the kinetics of these molecules *in vivo*, while addressing safety, stability and manufacturing issues. Combinations with adjuvants or other TLR agonists as well as optimized administration protocols should support research in this direction. Field studies with larger cohorts of animals will provide further insights on the utilization of CpG-A molecules for the prevention of viral infections in a clinical setting.

## Competing interests

The authors declare that they have no competing interests.

## Authors’ contributions

CRT and HL conceived and designed the project; HL supervised the study. CRT and VLR performed the experiments and analyzed the data with the support of VC, MLM and MF. BR was responsible for housing and care of the SPF cats as well as organization of blood collections. ME enabled work with Vesicular Stomatitis Virus. RHL, MF, ME, MT and PM provided crucial reagents, materials and/or analysis tools as well as valuable recommendations for their utilization. All authors critically revised the manuscript and approved the final version.
